# Viral Aggregation: The Knowns and Unknowns

**DOI:** 10.3390/v14020438

**Published:** 2022-02-21

**Authors:** Swechchha Pradhan, Arvind Varsani, Chloe Leff, Carter J. Swanson, Rizal F. Hariadi

**Affiliations:** 1 Center for Molecular Design and Biomimetics, The Biodesign Institute, Arizona State University, Tempe, AZ 85287, USA; carterswanson@gmail.com; 2 The Biodesign Center for Fundamental and Applied Microbiomics, Center for Evolution and Medicine, School of Life Sciences, Arizona State University, Tempe, AZ 85287, USA; Arvind.Varsani@asu.edu; 3 Structural Biology Research Unit, Department of Integrative Biomedical Sciences, University of Cape Town, Rondebosch, Cape Town 7700, South Africa; 4 School of Molecular Sciences, Arizona State University, Tempe, AZ 85287, USA; cleff@asu.edu; 5 Department of Physics, Arizona State University, Tempe, AZ 85287, USA

**Keywords:** viral aggregation, multiplicity of infection, viral transmission, viral infectivity

## Abstract

Viral aggregation is a complex and pervasive phenomenon affecting many viral families. An increasing number of studies have indicated that it can modulate critical parameters surrounding viral infections, and yet its role in viral infectivity, pathogenesis, and evolution is just beginning to be appreciated. Aggregation likely promotes viral infection by increasing the cellular multiplicity of infection (MOI), which can help overcome stochastic failures of viral infection and genetic defects and subsequently modulate their fitness, virulence, and host responses. Conversely, aggregation can limit the dispersal of viral particles and hinder the early stages of establishing a successful infection. The cost–benefit of viral aggregation seems to vary not only depending on the viral species and aggregating factors but also on the spatiotemporal context of the viral life cycle. Here, we review the *knowns* of viral aggregation by focusing on studies with direct observations of viral aggregation and mechanistic studies of the aggregation process. Next, we chart the *unknowns* and discuss the biological implications of viral aggregation in their infection cycle. We conclude with a perspective on harnessing the therapeutic potential of this phenomenon and highlight several challenging questions that warrant further research for this field to advance.

## 1. Introduction

Although there are no standard definitions of a viral aggregate, historically, it has been used to refer to multi-unit structures such as assemblages of viruses belonging to either the same or different species/families. Over time, researchers have documented such multi-unit structures using different terminologies depending upon their composition and the spatiotemporal context of their occurrence. For instance, outside the host, the insect-infecting baculoviruses (family *Baculoviridae*) [1] and ascoviruses (family *Ascoviridae*) [2] are embedded within highly organized crystalline protein lattices called occlusion bodies (OBs). OBs confer viruses with stability and resistance against adverse environmental conditions for an extended period, particularly considering their host’s cyclic and seasonal nature. Additionally, the OBs serve as transmission vehicles that facilitate the host-to-host transfer of multiple virions at once. Similarly, several early studies have reported aggregates of influenza virus (family *Orthomyxoviridae*) [3], vaccinia virus (family *Poxvirus*) [4], poliovirus (family *Picornaviridae*) [5], reovirus (family *Reoviridae*) [5], adenovirus (family *Adenoviridae*) [6], and rotavirus (family *Reoviridae*) [7], primarily in the context of the virus production processes or as occurrences in environmental settings that are relevant to human health. Similarly, several animal-infecting RNA viruses, such as enterovirus (family *Picornaviridae*) [8], rotavirus, and norovirus (family *Caliciviridae*) [9], have been reported to shed inside extracellular vesicles (EVs), like exosomes and microvesicles, in multiple numbers. These viruses can hijack the host extracellular vesicle biogenesis machinery to facilitate their collective assembly, envelopment, and subsequent dissemination through a nonlytic pathway. Finally, tetherin, an interferon-inducible host protein, has been identified as an antiviral factor that inhibits the release of a variety of enveloped viruses from the host cells [10,11,12,13]. More recently, Sanjuan coined “collective infectious units” as an umbrella term encompassing several types of structures that mediate the collective delivery of multiple virions/viral genomes to the same cell and often modulate viral infectivity differently than free viruses [14]. Some of these structures, including polyploid virions, occlusion bodies, viral aggregates, and lipid cloaked virions, are described in greater detail in another review [14]. Given the context of this review, we have used the term *viral aggregate* to refer to multi-unit structures comprising two or more virus particles without discriminating against their composition or causative factors responsible for their assembly.

In this review, we begin by summarizing the *knowns* of viral aggregation by providing a brief synopsis of historical studies that rather one-dimensionally focused on physicochemical parameters surrounding viral aggregation. We discuss the challenges faced by earlier studies and, concurrently, the limitations in our current knowledge of viral aggregation. We then explore the *unknowns* and expand the dimensionality of the field by discussing viral aggregation in light of viral pathogenesis. We critically assess a few studies that provide direct evidence of how viral aggregation affects their infectivity in simulated biological models. Next, to consider the implications of viral aggregation in the broader and largely ignored context of an infectious viral life cycle, we review studies providing correlations between viral aggregation and one or more components of their life cycle. We conclude by shedding light on the therapeutic potential of viral aggregation and formulating several challenging questions that need further investigation for this field to advance.

## 2. A Brief Historical Review of Studies on Viral Aggregation

A quick survey of available literature on viral aggregation reflects its fascinating scientific journey. More than eight decades ago, the first studies reported aggregates of plant-infecting tobacco mosaic virus (TMV, family *Virgaviridae*) [15] and animal-infecting influenza viruses [16] merely as undesirable technical artifacts causing inconsistencies in viral titers and the serum titers required to neutralize them. Studies that came in the 50s and 60s reported how aggregation compromised the “quality” of laboratory-propagated strains of different viruses by reducing their infectious titers as assessed by plaque assays [4,17]. In the environmental context, aggregates of poliovirus, reovirus, and adenovirus caused problems in water decontamination processes because of their enhanced resistance to disinfectants in comparison to monodispersed particles [18,19,20]. In the two decades that followed, scientific research mainly aimed at preventing or disintegrating viral aggregates to increase the infectious titer of laboratory-grown viral strains, minimize their batch-to-batch variations, and enhance the efficiency of virus neutralization in vitro and disinfection processes in the environment. These studies investigated the physicochemical parameters influencing viral aggregation and subsequently underscored its role in viral transport, adsorption, and retention, mainly in water bodies and in vitro settings [5,20,21,22,23,24,25,26,27]. We have summarized them in the following two sections of this review.

A few other studies in the 60s and 70s showed the ability of aggregated vaccinia viruses and influenza viruses to overcome genetic defects and enhance their infectivity and survival in cultured cells [3,28]. For instance, the survival curves for vaccinia viruses showed a slower decline in their titers upon UV irradiation when they were in an aggregated state compared to their monodispersed form [28]. Another study showed that aggregation increased the infectious virus titer per genome and rescued the infective potential of mutant influenza viruses with defective genomes [3]. These studies established a potential correlation between viral aggregation and multiplicity reactivation—a phenomenon by which viable viruses are released from cells infected by two or more viruses, each with a uniquely defective genome.

It is important to note that for a long time, viral aggregation was only studied as a consequential phenomenon, with the main determinants being the physicochemical interactions virus particles have at different interfaces. A few studies in the 1980s started challenging this perspective when they reported membranous aggregates of several pathogenic viruses in fecal specimens of patients with gastroenteritis [6,7]. The electron microscopy (EM) images of some of these viruses are given in Table 1. The observed aggregates were neither technical artifacts nor seemed to result from interactions with other biomolecules such as cell debris, proteins, or antibodies. They postulated that these aggregates formed during virus maturation or assembly inside host cells [7]. These studies led the scientific community to question if aggregation could also be an intrinsic viral trait that happens during the viral infection cycle and influences their infectivity and pathogenicity. With the advances made in live-cell imaging and molecular biology, an increasing number of studies have reported the aggregation of different animal-infecting viruses with potential implications in their infectivity, fitness, and evolution. We have discussed them in separate sections of this review. However, since this field of research is relatively new, there is a lack of standard guidelines and definitions for better describing and differentiating viral aggregates.

It is safe to assume that the analyses in many historical studies were technologically challenged, because of which their appeal could be limited to current virologists. Nevertheless, these were fundamental concepts that laid the foundations for many of the basic laboratory practices in virology followed to date. Furthermore, these studies highlighted that viral aggregation could have crucial public health and biotechnology implications by providing insight into how the virus production processes in laboratory settings emulate natural environments and into the stability of viable viruses following groundwater transport and wastewater treatments.

### 2.1. Factors Influencing Viral Aggregation

The interplay between viruses and different biotic and abiotic factors present in their microenvironment plays a vital role in viral aggregation. In suspension, viral aggregation is affected by several physicochemical parameters of the aqueous medium, including but not limited to pH, ionic strength and composition, and temperature. Some studies investigating these parameters have shown that aggregation can be reversible for some viruses. For some viruses, these parameters also govern the degree of reversibility of viral aggregation [5,21,24,32].

Viruses get their net charge from different functional groups present in their phospholipid envelope or capsid proteins. Their isoelectric point (pI) ranges from 1.9 to 8.4 [33], and they tend to aggregate near their pI, where their net neutral charge cancels the electrostatic repulsion between particles [34,35,36]. Lowering the pH also favors viral aggregation and, for some viruses, this could recapitulate the acidic conditions inside endosomes that trigger their uncoating and subsequent release into the cytoplasm [37,38]. Likewise, ionic strength and composition affect viral aggregation by compressing or expanding the electric double layer (EDL) surrounding viral particles [34,35,39,40].

Cations and cationic polymers complex with the exposed and deprotonated carboxylic groups of polar amino acids on the viral surface and reduce their zeta potential [40]. Divalent cations aggregate viral particles more strongly than monovalent cations due to increased charge shielding and EDL shrinking [34,35,40]. On the other hand, anions and anionic polymers can add to the EDL and increase the zeta potential by sequestering cations and adsorbing to the viral surfaces, preventing charge shielding and virus aggregation [34,39].

Similarly, temperature influences viral aggregation, with more aggregates forming at higher temperatures. Viruses are colloidal particles, and as such, their Brownian motion and subsequent collision rate rise at higher temperatures leading to more aggregation. Several studies exploring the kinetics of viral aggregation as a function of these physicochemical parameters show that the process is mainly virus-specific and depends on the surface properties of viral particles. For instance, four genogroups of F-specific RNA bacteriophages, MS2, GA, Qβ, and SP, showed different aggregation behaviors over a broad range of pH (1.5–7.5) and ionic strength (1–100 mM NaNO${}_{3}$) conditions tested [41]. While MS2 only aggregated near their isoelectric point (pH = 4) regardless of the ionic strength, Qβ aggregated at low pH and high ionic strength, and GA and SP both aggregated over the entire range of tested conditions.

Most earlier studies investigated these parameters from a technical standpoint, either to improve the monodispersity of viral particles in purified viral stocks or to improve the inactivation kinetics of viruses in different settings. They have been discussed more extensively in another review [42]. A key challenge for this expanding field of scientific study is to link these primarily in vitro described environmental parameters to those that are likely to be encountered within the viral life cycle in a host. Studies have shown that viral aggregates are a non-negligible fraction of their populations and maybe more frequently prevalent in biological fluids than estimated or known in vitro [9,43]. Therefore, these findings will be critical in determining the nature of interventions needed to contain or eliminate viruses, particularly for those causing the emergence/re-emergence of infectious diseases. Some of the recent work has begun to address that challenge by exploring the aggregation of vesicular stomatitis virus (VSV, family *Rhabdoviridae*) under physiologically relevant conditions [43,44]. VSV is an economically significant livestock virus that primarily infects the oral cavity and is shed in the host saliva. Microscopic analysis of VSV-infected cultures showed two phenotypically distinct genetic variants of VSV, one expressing mCherry and the other expressing GFP, aggregated in the presence of human and cow saliva [43]. Another work revealed that unlike protein–lipid interactions driving VSV aggregation in purified stocks [43], VSV aggregation in saliva was protein-driven [44]. Proteomic analysis revealed the differential expression of 18 different genes among saliva donors that positively correlated with their aggregating potential. Furthermore, fibrinogen gamma chain (FGG) protein was identified as the molecular factor strongly promoting VSV aggregation in saliva. For most other infectious viruses, the physicochemical and molecular determinants of viral aggregation in environments recapitulating viral hosts remain to be investigated.

### 2.2. The Research Landscape of Viral Aggregation in Comparison to Their Bacterial Counterparts

Analogizing viral aggregation to its bacterial counterpart, aggregation in bacteria has been rather extensively studied. Bacterial aggregation is a corollary defense mechanism against environmental stress and immunological response [45,46,47]. Distinct genetic processes regulate bacterial aggregation in response to stress factors such as harsh environmental conditions and attacks from predators, primarily bacteriophages. Studies have shown multiple bacterial species including *Escherichia coli* [48], *Pseudomonas aeruginosa* [49], *Legionella pneumophilia* [50], *Staphylococcus aureus* [51], and *Neisseria meningitidis* [52] converge on this strategy. For a more in-depth insight into this area, we refer readers to another review discussing molecular mechanisms underlying bacterial aggregation and its role in bacterial pathogenesis [53].

In contrast, the genetic and molecular mechanisms driving viral aggregation are largely unresolved and unfortunately understudied. Although several studies claim viral aggregation as an intrinsic phenomenon, our understanding of its role in viral population dynamics and evolution is limited. What is striking from the reviewed literature is that aggregation spans across various viral families, including enveloped and non-enveloped viruses, segmented and non-segmented viruses, and DNA and RNA viruses. So, aggregation may be more broadly common than is known and potentially with a fitness advantage to some viruses.

## 3. Viral Aggregation and the Stoichiometry of MOI

Viral populations encounter several viral factors and host barriers as bottlenecks during their infection cycle in the context of both intra- and inter-host transmission. As obligate intracellular parasites, viruses depend entirely on the host cell and use a “Trojan horse” machinery to encode viral proteins and replicate their genetic material. The multiplicity of infection (MOI) is the most critical parameter affecting their infection cycle at the cellular level. MOI, defined as the ratio of infectious virions to susceptible cells, controls the gene copy number and determines the fate of the infected cell. An MOI higher than 1 sets the stage for genetic exchange [54], competition [55], or complementation [56] to occur between co-infecting viral genomes. Viral aggregation is associated with increasing MOI and the subsequent co-transmission of multiple viral genomes to the same cell [4,6,7,28,40,57]. Therefore, it is essential to study how it can regulate these phenomena and impact the broader viral pathogenesis, fitness, diversity, and evolution spectrum.

### 3.1. The Stochasticity in Early Events of Viral Infection Often Leads to Unproductive Infection

Historically, the “one-hit” paradigm in virology views viral particles as independent and optimal infectious units such that one infectious unit is enough to establish a productive infection [58]. According to this theory, at high dilutions of virus particles, one infectious particle gives rise to one plaque, and the number of plaques is directly proportional to the concentration of the virus. Most mammalian viruses show a linear relationship between the number of plaques and dilution of virus plated, holding the framework true in titration assays to determine viral titers and the infectious dose. However, one of the major limitations of this framework stems from its failure to address the stochastic fluctuations that challenge early events of viral infections and render most infections futile.

As with the vast majority of pathosystems, the mere existence of a virus in a suitable microenvironment with many permissible and susceptible host cells is not sufficient to guarantee successful infection. Viral infections are stochastic and discrete events, influenced by several viral factors and host barriers, which pose challenges of thwarted outcomes in each step of their life cycle. Several studies have reported cell-to-cell variability surrounding different phases of viral infections, including viral endocytosis [59], virus progeny titers, RNA levels [60], and progeny production modes [61]. The variability is attributed to noisy biochemical processes involved in viral infections. For instance, stochastic fluctuations accounted for up to 90% of failed single-hit infections with influenza A virus (IAV) [60]. Apart from different components of the host immune responses which can neutralize them, there are other mechanical, physicochemical, and genetic barriers for viruses to overcome, the permutations and combinations of which can further hinder their infection cycle.

### 3.2. Segmented and Multipartite Viruses Have Low Infection Probability

Although many RNA and DNA viruses show non-infectiousness and low infectivity, they are more prominent in viruses with either segmented genomes [62] or multipartite genomes [63,64]. Segmented viruses have the information required for the infection cycle divided between two or more nucleic acid segments, typically found together in one capsid. However, not all segments are needed for the virus to be infectious. For instance, influenza A virus (IAV) has eight single-stranded RNA segments, each encoding at least one viral protein [65,66,67]. Studies have shown that single-hit IAV infections predominantly failed to replicate and resulted in semi-infectious viral particles lacking one or more of the essential viral proteins [60,68,69]. Multipartite viruses are slightly different and instead have their genetic information divided into segments packaged into independent viral capsids [70]. The dose-response kinetics of Guaico Culex virus (GCXV, family *Flaviviridae*), a five-segmented RNA virus, showed that establishing a productive infection required at least three different particles [71]. Multipartite genomes underscore the interdependency of viruses in these systems, necessitating the co-transmission of several virus particles into one host cell to form a complete genome set and increase the likelihood of a productive infection. The cost–benefit analysis of these peculiar genome organization systems in viruses has posed some of the most exciting puzzles for virologists concerning the importance of virus genome integrity for successful infection cycles [60,71].

The stochastic, genetic, structural, and host barriers are magnified in low MOI or single-hit infections and may enable viruses to adopt mechanisms more conducive to preserving their genome integrity. Several studies have shown that aggregation increased the cellular MOI and enhanced viral infectivity [3,8,43]. The substantial body of work on collective infectious units in viruses describes several structural systems that support viral co-transmission, including polyploid virions, virion aggregates, viral occlusion bodies, and virions with extracellular vesicles [14,72]. All of these structures assist in increasing cellular MOI and subsequently delivering multiple viral genomes to the same cell, which can help in overcoming the replication barriers mentioned above.

## 4. Viral Aggregation in the Context of Infectious Viral Life Cycle

Viral aggregation impacts different aspects of viral pathogenesis including infectivity, antibody escape, and antiviral resistance. Some studies have demonstrated an enhancement of viral infectivity when viruses were in an aggregated state as opposed to being in a monodispersed state [3,9,73,74]. On the other hand, some studies have demonstrated that viral aggregation compromised the replication, transmission, and survival of viruses [75,76,77]. We have summarized the impacts of viral aggregation on the viral life cycle and pathogenesis in Table 2. To better understand the apparent discrepancy concerning the cost–benefit of viral aggregation, we have categorized the events in the life cycle of an infectious virus into three distinct stages, including viral motion to find a suitable host, replication inside the host cell, and release from the host cell. Aggregation may favor or oppose the infecting viruses in each stage, subsequently influencing their outcomes and determining their fate.

### 4.1. Viral Aggregation Influencing Viral Motion

**Figure 1:**
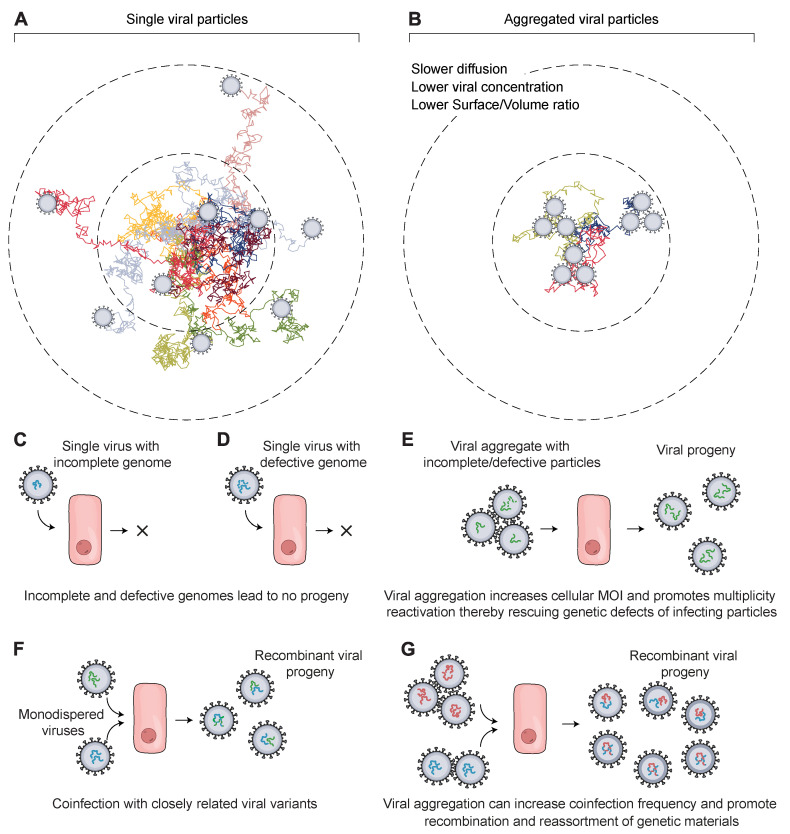
Schematic showing how viral aggregation affects their ability to infect target cells and their evolution. (**A**) A hypothetical arrangement of monodispersed viral particles (*N* = 9 viral particles) has a relatively faster diffusion, which increases their dissemination and the frequency of adsorption to target cells. (**B**) Aggregated viral particles (*N* = 3 trimers = 9 viral particles) diffuse more slowly and lead to a lower effective titer, which decreases their association rate with target cells before being deactivated or degraded. (**C**,**D**) In the case of multi-segmented and multipartite viruses, a single virus particle is likely to fail in producing progeny due to several challenges, the most prominent being defective or incomplete genomes. Following virus entry, the viral genome is released inside the host cell to start viral replication. However, the genome is highly likely to be incomplete or defective, particularly with RNA viruses such as influenza. This results in the failure of the virion to transcribe or translate necessary viral factors to produce infectious progeny. (**E**) Invasion of host cells by viral particles in an aggregated state is conducive to increasing cellular MOI, which releases multiple copies of the viral genome inside the host cell. This sets the stage for genetic complementation and multiplicity reactivation, which facilitates the overcoming of any genetic defect or missing genetic factors. It increases the chances of the virions to replicate and produce viral progeny that will start their infection cycle. (**F**,**G**) Genetic recombination and reassortment between closely related virions in either the monodispersed state (**F**) or the aggregated state (**G**) can produce chimeric progeny with genetic segments derived from each parent. This influences their fitness and contributes to genetic diversity.

Viruses are colloidal particles with defined densities and intrinsic half-lives. Owing to the rapid decay rate of viruses, it is a race against time for them to find permissible host cells before they start degrading [87]. Devoid of locomotory organs, viruses travel through diffusion in their microenvironment to establish initial contact with host cells and start their infection cycle. Aggregation slows down diffusion, decreases the surface-to-volume ratio, and lowers the number of effective viral particles (Figure 1A,B). In addition, it reduces the frequency of viral adsorption onto host cells and their likelihood of reaching the maximum number of host cells.

To traverse the distance to their host cells before degradation, they rely on their Brownian motion and the movement of their surrounding fluid. Upon breaking down the effects of aggregation on the viral life cycle, we think the distribution of viral particles will be consequential based on the following rationale despite this not being tested experimentally. The mean squared displacement $\langle \Delta r^{2}\rangle$ of viral particles over time *t* is defined by the equation of mean squared displacement (MSD) for a three-dimensional Brownian motion, given by

(1) $$\langle \Delta r^{2}\rangle =6Dt,$$

where *D* is the diffusion coefficient. Assuming a viral particle/aggregate as a sphere of an effective radius *r*, the diffusion coefficient of the viral assembly is described by the celebrated Stokes–Einstein equation,

(2) $$D=\frac{k_{B}T}{6\pi \eta r},$$

where $k_{B}$ is the Boltzmann constant, *T* is the absolute temperature in Kelvin, and η is the viscosity of the medium. For example, the diffusion coefficient of a single IAV particle has been measured to be ∼800 nm${}^{2}$/s [88] with a half-life of ∼3 h [89]. Using these numbers, Equation (1) yields ∼7 μm for the average displacement after 3 h. The calculated mean-squared displacement is less than the typical size of a mammalian cell (10–100 μm). Diffusion is, therefore, one of the limiting factors determining the success of the earliest events in infection. Viral particles with a size of less than 100 nm can form aggregates that are up to 1000 nm [6]. Regarding the excursion to reach the host cell, monodispersed particles diffuse faster than the aggregated particles, and so they will collide with the host cell surfaces more frequently. Assuming host cells as uniform spheres of radius *a* and invoking a diffusion-limited reaction, the number of viral particles arriving per unit of time is given by

(3) $$\frac{dN}{dt}=4\pi Dac$$

where *c* is the concentration of the viral monomers/aggregates. Moreover, the effective number of viral particles reaching more host cells is higher when they are in a monodispersed state than when they are in an aggregated state by a factor of N/n, where *n* is a positive integer and denotes the mean size of the viral aggregates. Viruses with a shorter half-life will reach even a smaller number of host cells before decay. The poor transduction efficiency of retroviruses has been attributed to their short half-life, limiting the distance they can travel in solution by Brownian motion [90].

Once virus particles adsorb on the surface of a suitable host, they begin the multistep and tightly controlled process of entering the host cell. It starts with the virus binding to specific receptors or attachment factors such as carbohydrates, lipids, and other cellular proteins on the host cell surface [91]. After binding, they enter host cells either by endocytosis or by direct fusion with the host cell plasma membrane. However, regardless of the route taken, the end goal is to release viral genomes in the cytoplasm, where they are processed further for nuclear import [92]. For many years, scientists seeking to decipher the molecular mechanisms driving viral entry studied singular interactions between a virus and a host cell but largely ignored viral aggregates. The dynamics of viral entry for viral aggregates will likely be different than for a single virus particle and depend on the aggregate’s size, shape, and composition. However, they remain yet to be investigated and are far from resolved.

### 4.2. Viral Aggregation Influencing Replication Inside Host Cells

Following cell entry, viral genomes are transported to the nucleus or specific sites in the cytoplasm for replication, expression of viral proteins, and assembly [92]. Viral aggregates diffuse more slowly and are likely to infect fewer cells than their monodispersed counterparts. However, for some viruses, aggregation compensates this cost by increasing the MOI, subsequently reducing the risk of stochastic failures. For instance, cells infected with saliva-induced aggregates of VSV and phosphatidylserine (PS)-enclosed aggregates of enteroviruses produced higher progenies than cells infected with an equal number of monodispersed viruses [8,73]. Microscopic analyses of cells infected with VSV aggregates and enterovirus aggregates in these studies showed the transmission of multiple viral genomes to the same cells. Interestingly, VSV aggregation did not compromise their dispersal capacity, and the higher MOI did not rescue genetic defects [73]. Instead, the fitness advantage of VSV aggregates correlated with cellular permissivity to infection and the increased chances of overcoming initial stochastic barriers. On the other hand, like many other RNA viruses, enteroviruses have high mutation rates and exhibit a great deal of genomic heterogeneity. The enhancement of the replication kinetics of vesicle-enclosed enterovirus aggregates correlated with genetic complementation, reductions in stochastic fluctuations, and the PS-mediated enhanced modulation of antiviral response [8]. In another recent study, vesicle-enclosed aggregates of rotaviruses showed enhanced infectivity in vitro and in vivo in mice compared to freely dispersed viruses [9]. As causative agents of gastroenteritis, rotaviruses infect the intestinal cells and transmit through the fecal-oral route. In this study, vesicle-enclosed rotaviruses overcame the intrinsic replication barrier of RNA viruses by ensuring a more concentrated delivery of viral particles and enhanced their infectivity by providing a higher degree of protection from host immune components as viruses traverse through the GI tract before infecting the intestinal cells.

In addition, during replication, the presence of multiple viral genomes can promote genetic interactions such as recombination, competition, and complementation. These interactions can influence viral fitness, diversity, and evolution (Figure 1C–G). In an early line of work, aggregates of UV irradiated vaccinia viruses showed enhanced survival compared to monodispersed viruses [28]. For RNA viruses, the impact of these interactions could be even more profound. Because of the lack of proofreading activity of their RNA-dependent RNA polymerases, they have high mutation rates and often fail to establish productive infections (Figure 1C,D). For instance, about 90% of influenza viruses failed to express one viral protein [68]. A higher MOI may promote complementing and cooperative interactions among viral genomes, rescuing their lethal/defective mutations and enhancing their infectivity [3,60].

### 4.3. Viral Aggregation Influencing Release from Host Cells

In the canonical route of virus release, enveloped viruses leave the infected cell by budding and secretion [93]. Non-enveloped viruses typically lyse the host cells to exit them. However, some of them escape via secretory pathways. They can bud into intracellular multivesicular bodies (MVB) and leave after fusing with the plasma membrane. Some follow the non-canonical route, subverting cellular autophagy and releasing by secretory mechanisms.

According to the conventional model of viral transmission, viral particles release and spread as free individual particles, and the fate of individual viral genomes is not inter-dependent during virus trafficking [94]. This concept has been contended by several lines of work, which are discussed in the following sections. Some viruses converge inside or on the host cell surface to form multi-virion structures before release. These structures can modulate vital aspects of viral pathogenesis, including infectivity, virulence, transmission, antibody escape, and fitness.

#### 4.3.1. Extracellular Vesicles-Mediated Release of Viral Aggregates

**Figure 2:**
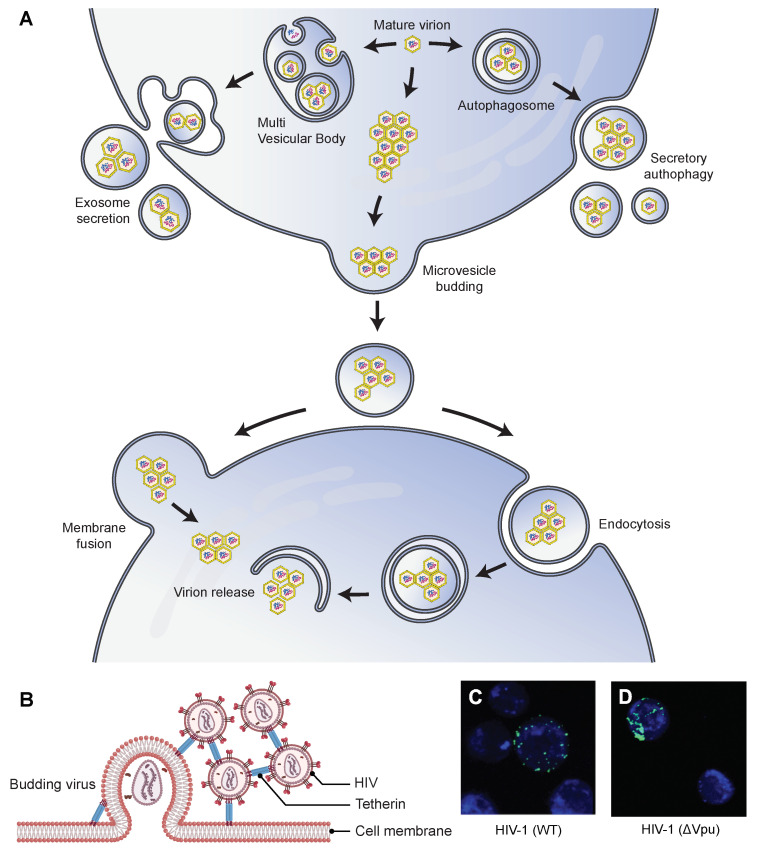
Possible mechanisms by which the aggregation of virus particles affects their transmission ability. (**A**) Virions can aggregate and be subsequently released from their host cells inside extracellular vesicles (EVs). They can aggregate inside microvesicles that are released directly from the plasma membrane using a budding mechanism. They can also bud into multivesicular bodies (MVB) that are trafficked to the plasma membrane and released into the extracellular space by membrane fusion. They can also aggregate inside autophagosomes and be released using the secretory autophagy pathway. After release, the EV-enclosed virions can enter new host cells either by fusion at the cell membrane or by the endocytic route. EVs enhance the transmission ability and the subsequent infectivity of virions by protecting against neutralizing antibodies [74] and promoting the collective delivery of multiple virions [8,74], respectively. (**B**) Schematic representation of tetherin (an interferon-inducible antiviral factor)-mediated aggregation and retention of HIV particles on the surface of the infected cells, which affects the cell-to-cell transmission of the virus. Tetherin colocalizes with Gag protein at the plasma membrane and is antagonized by Vpu protein. (**C**,**D**) Correlative light-scanning electron microscopy (SEM) images showing the distribution of HIV-GagGFP (WT or ΔVpu) particles (green) on target Jurkat cells (blue) [75]. Cells were harvested after 2 h of cocultivation with WT or ΔVpu HIV-transfected HeLa donor cells. In the presence of Vpu, WT HIV particles were transferred as small clusters (**C**), and in the absence of the antagonist, ΔVpu HIV particles were transferred as larger aggregates (**D**). Parts (**C**,**D**) are republished with permission from [75]. Copyright 2010 under Creative Commons Attribution License 2.0.

In addition to being carriers of biomolecules (nucleic acids, proteins, lipids) and mediums for cell–cell communication, extracellular vesicles (EV) can also carry virus clusters and function as independent infectious units [93]. The EV-mediated transfer of viral clusters is termed as vesicle-mediated en bloc transmission [93]. Several recent findings showed EV-mediated in vitro release and transmission of clustered enterovirus [8,86,95], hepatitis A viruses (HAV, family *Picornaviridae*) [74], rotavirus and norovirus [9]. Some of them clustered within phosphatidylserine (PS) lipid-enriched vesicles [8,9]. Following the common routes of EV biogenesis, vesicle-enclosed virus clusters can originate intracellularly from autophagosomes and multivesicular bodies (MVBs) or directly from the host cell plasma membrane [74,86,95]. However, vesicle-enclosed viruses always follow the non-lytic mode of virus release, blurring the conventional distinction between enveloped and non-enveloped viruses. A schematic representing different routes of EV-mediated viral release is shown in Figure 2A. A recent review has discussed the advantages of EV-mediated en bloc transmission of several infectious viruses along with the known molecular mechanisms of cargo delivery [96].

Poliovirus demonstrated the lysis-independent release of viral clusters within host-derived vesicles [86]. Quantitative single-cell analysis showed the virus clusters originated from autophagosomes. However, viruses subverted the autophagy pathway by inhibiting the fusion of autophagosomes with lysosomes, followed by their non-lytic release in single-membrane vesicles. This process is called *autophagosome-mediated exit without lysis* (AWOL) [97]. Upregulation of the autophagy pathway enhanced viral spread in vitro and pathogenicity in mice. In another work, Hepatitis A viruses (HAV, family *Picornaviridae*) demonstrated AWOL-mediated non-lytic release from exosome-like EVs. The vesicle-enclosed viruses showed enhanced infectivity and resistance against antibodies [74]. The formation of these extracellular vesicles relied on the multivesicular body (MVB) components and the autophagy pathways. In another work, the sequential events of infection and viral spread of coxsackievirus B3 (CVB3) were tracked in real-time using a recombinant virus, Timer-CVB3, which expressed a fluorescent timer protein that changed color from green to red over time. The progression of Timer-CVB3 in partially differentiated neural progenitor and stem cells (NPSCs) revealed that the viruses frequently pooled together inside extracellular microvesicles (EMVs) and released in a lysis-independent manner [95]. The study postulated that the EMV-mediated release of viral clusters could enhance viral spread by exploiting the migratory nature of progenitor cells and modulating cellular differentiation to catapult viral egress in the absence of cell lysis.

#### 4.3.2. Tetherin Mediated Viral Aggregation and the Consequent Inhibition of Viral Release

A rather intriguing route of viral aggregation is mediated by tetherin, an interferon-induced cellular restriction factor that acts as an innate antiviral defense against HIV [10,75] and other enveloped viruses, including other retroviruses [98], filoviruses [98], gamma-herpesviruses [99], and rhabdoviruses [100]. Mutational analyses have revealed the autonomous mode of tetherin function is determined by its overall configuration rather than sequence homology [10].

In the case of HIV, tetherin accumulates with viral Gag proteins at cell surfaces. It incorporates itself into assembling virions as a disulfide-linked dimer using either of its two membrane anchors [10]. This simple configuration of tetherin directly tethers virion particles to the cellular membranes of infected cells and retains them (Figure 2B). In response, viruses have also adapted mechanisms to interact with tetherin to impede its function. For instance, the HIV-1 accessory protein, Vpu, acts as a viral antagonist of tetherin [101].

A few studies have shown the tetherin-mediated aggregation and retention of HIV, however, with different implications on the cell-to-cell release of viruses [75,80,102]. In general, mature virions can employ any of the several routes for direct cell-to-cell transmission, including viral synapses, polysynapses [103], filopodial bridges [104,105], and viral biofilms [81]. FACS analyses showed tetherin inhibited the cell-to-cell transfer of HIV from infected donor cells to uninfected target cells [75,102]. Casartelli et al. showed that upon infection, tetherin-expressing cells transferred HIV aggregates as abnormally large patches (Figure 2C,D) that were impaired in their fusion capabilities [75]. In addition, target cells showed lower levels of viral DNA over time when co-cultured with tetherin-expressing donor cells infected with Vpu-defective HIV (ΔVpu). Conversely, Jolly et al. showed that tetherin expression enhanced the cell-to-cell transfer of viruses, most likely by increasing the localized and effective concentration of virions [80]. Contrary to the previous work, viral DNA synthesis in target cells co-cultured with ΔVpu HIV-infected donor cells increased over time. The increase was not as rapid in target cells co-cultured with WT HIV-infected donor cells, implying enhanced transmission of ΔVpu HIV. In addition, tetherin inhibition did not increase viral spread, and the tethered virions remained fully infectious. While the implications of tetherin-mediated retention of viruses on viral transmission need further investigation, the contrasting findings in these studies potentially reflect the dynamic nature of tetherin modulation that depends on cell type and expression level of other cellular and immune components.

## 5. Viral Aggregation as an Antiviral Response

Host immune responses present a significant barrier for viruses. Throughout their infection cycle, they encounter different components of the immune system, ready to neutralize any incoming pathogen. Depending on the nature of the viral infection, it may activate various components of either the innate immune system or the adaptive immune system or both [106,107,108]. Innate immune responses are rapid but largely non-specific. As the first line of defense, they neutralize infiltrating viruses directly by macrophage and neutrophil-mediated phagocytosis and indirectly by natural killer cell-mediated apoptosis or complement-mediated lysis. If some viruses evade innate responses, the adaptive immune system kicks in. The adaptive response relies on antigen-presenting cells (APCs), such as dendritic cells and macrophages, to successfully activate cytotoxic T cells (CTLs) that kill infected cells and B cells that synthesize virus-specific antibodies. The distinguishing feature of adaptive immunity is its ability to differentiate between non-self-materials, leading to the development of immunological memory, which causes the immune system to respond more vigorously to re-exposures.

Several lines of evidence suggest that viral aggregates are more resistant to chemical disinfection and antibody neutralization [22,26,74,96,109]. Vesicle-enclosed viral clusters, in particular, can modulate host responses to enhance their infectivity in different ways [96]. Here, we discuss viral aggregation as a common antiviral host response mechanism.

Aggregation is a standard route taken by antiviral agents to neutralize viral infections. We have highlighted some studies showing the aggregation of influenza A virus (IAV) following their interaction with different antiviral components in Table 3. For instance, natural IgM and the complement system worked synergistically to neutralize viral particles primarily by aggregating them [110]. The IgM-mediated deposition of complement proteins on the viral surface aggregated viruses and subsequently neutralized them by blocking the accessibility of hemagglutinin (HA) receptors for their cellular ligands. HA glycoproteins coordinate the effective membrane fusion of influenza viruses with the host cells. In several other lines of work, soluble innate inhibitors, such as lectin inhibitors and antimicrobial peptides, aggregated viruses and neutralized them [82,83,111,112,113,114,115]. The neutralizing potential of antimicrobial peptides correlated with their aggregating potential [82]. Aggregation reduced the effective virus concentration, promoted their clearance from the airway through mucociliary action, and enhanced phagocytosis. In another study, histone proteins neutralized H3N2 and H1N1 influenza viruses by aggregating them directly and inhibiting their internalization [84]. The arginine-rich histone, H4, had the most potent anti-influenza activity of all core histones tested. In another study, a 20 amino acid EB peptide aggregated H5N1 influenza viruses, resulting in reduced virus binding with host cell receptors and increased opsonization [76]. Incorporating the peptide as adjuvants in H5N1 vaccines reduced influenza-associated morbidity in mice and enhanced viral clearance by improving cell-mediated immune response. These studies set a precedent for harnessing viral aggregation as a tool to develop novel antiviral therapeutics.

## 6. Harnessing Viral Aggregation as a Therapeutic Tool

**Figure 3:**
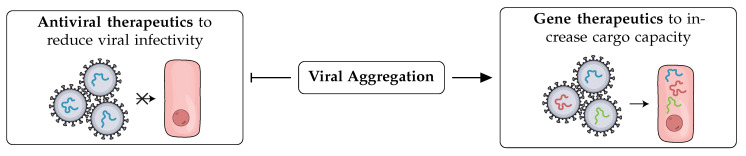
A viral aggregation strategy can potentially be harnessed to decrease viral infectivity (**left**) or to increase cargo capacity and subsequent transduction efficiency of viral vectors (**right**). Viral aggregation can be induced by introducing multivalent viral binders or by modulating their environment.

Given the pervasive impact of aggregation on the life cycle, fitness, and pathogenicity of infectious viruses, we cannot neglect the potential of harnessing this phenomenon as a therapeutic tool. Viruses were discovered as infectious agents and repurposed as gene delivery vehicles over time. Infectious disease research is heavily focused on developing robust and rapid antiviral therapeutics. At the same time, gene therapy studies put considerable effort into engineering viral vectors with higher cargo capacity, inert immunogenicity, and strong transduction efficiency. We understand that the phenomenon of viral aggregation can be repurposed to cater to both dimensions of research focusing on viral infections (Figure 3).

Several lines of evidence show that aggregation is a standard route taken by antibodies and antivirals to neutralize viruses [82,83,112,114,119]. Aggregation slows down the diffusion of viral particles and reduces their effective concentrations. Bound by their intrinsic decay rates, aggregated viruses are likely to infect fewer cells than freely dispersed viruses. Therefore, an effective antiviral strategy could be a platform that aggregates viral particles and minimizes their interactions with host cell surfaces.

The synergy between nanomaterials and small molecules (proteins, peptides, aptamers, etc.) has been increasingly exploited to develop nano-enabled solutions that address modeling, diagnostic, and therapeutic challenges in various viral pathosystems. Similar design principles can be used to fabricate nanoscale platforms that aggregate viral particles, subsequently limiting viral diffusion and adhesion onto the host cell surface. For instance, two-dimensional and three-dimensional nanostructures that can cross-link circulating viral particles could be a logical design to aggregate viral particles. Similarly, interfacial nanostructures enabling the physical entrapment of circulating viral particles could also be a potential platform design to aggregate viral particles. Synthetic peptides [76,120], nucleoside analogs, proteins [121], and nucleic acid aptamers [121] can be chemically conjugated as virus binders to a wide variety of biocompatible nanomaterials (DNA-based, carbon-based, polymers, dendrimers, etc.) that can provide the structural framework/backbone to cross-link or entrap viral particles. Unlike many antivirals that target cellular mechanisms, such platforms can directly target viruses and function autonomously. For instance, the potential of IAV-aggregating EB peptide to work as a vaccine adjuvant has been previously established [76]. The same molecule can be incorporated into multivalent nanostructures and repurposed as an antiviral that aggregates multiple viral particles at once.

Viral aggregation can also be leveraged to engineer enhanced gene delivery vehicles for gene therapy. Viral vectors are the gold standard for in vitro and in vivo gene delivery. Adeno-associated virus (AAV) vectors, with their diverse tissue tropism and low immunogenicity, are the leading gene delivery platforms for gene silencing, editing, and replacement therapeutics [122,123,124]. However, their therapeutic applications are limited mainly because of their small cargo capacity (4.7 kb). Many studies have focused on engineering the AAV genome and capsid to enhance gene delivery efficiency with minimum immunogenicity. Scientists have developed the split AAV vector approaches that enable the delivery of genetic fragments larger than 4.7 kb [125,126]. These systems utilize genome fragmentation, overlapping, and trans-splicing mechanisms to divide the transgene into multiple fragments and rely on genetic cues post vector co-infection to regenerate the entire transgene. For instance, one study used an overlapping strategy to fragment the alkaline phosphatase gene into two AAV vectors and deliver the gene to airway epithelial cells in mice [125]. Another study used a trans-splicing vector approach to fragment a 6 kb mini-dystrophin gene into two AAV vectors and deliver it to a mouse model of muscular dystrophy [126]. The interdependency between AAV vectors presents a major limitation in these systems. The complete functionality of the transgene within a cell is contingent upon the co-delivery of all AAV vectors in the same cell. Therefore, it is challenging to realize the potential of these platforms until they incorporate modalities to guarantee the co-delivery of all AAV vectors. This gap can be addressed by nano-enabled platforms immobilizing viruses such that the delivery of the platform guarantees co-infection of all viruses. For instance, it is possible to design platforms that can integrate multiple AAV vectors into one functional unit for cellular delivery. Each AAV vector could carry a fragment of the desired transgene or a component of the multi-unit genome editor (for instance, either gRNA or Cas9 or fusion proteins in the context of CRISPR-Cas9). Cells infected with these viral assemblies would, in principle, have higher co-infections and subsequently better chances at reassembling all the fragments and producing the full-length transgene.

## 7. Concluding Remarks and Prospects

Viral aggregation is a widespread phenomenon affecting different aspects of viral infectivity, survival, and population dynamics. In the initial stages of infection, it can hinder viral spread by limiting the diffusion of viral particles. However, it can compensate for the loss by increasing cellular MOI, reducing stochastic barriers, and enhancing infectivity. In addition, a higher MOI sets the stage for genetic interactions among co-infecting viruses, with potential implications in viral diversity and evolution. Vesicle-enclosed viral aggregates act as optimal infectious units, mediating non-lytic release, en bloc transmission of viruses, and enhanced immune evasion. Aggregation is also the main route taken by antibodies and antiviral compounds to neutralize viruses, and as such, viruses aggregated by antivirals show enhanced opsonization and rapid clearance. However, these outcomes are not absolute and vary depending on the viral species and the spatiotemporal context of viral aggregation.

Live-cell imaging studies coupled with single virus tracking have provided more profound insights into molecular mechanisms underlying virus infection, trafficking, and interactions with cells, antibodies, and antivirals. However, for aggregated viruses, these molecular mechanisms are far from resolved. Given the impact of viral aggregation on different aspects of viral infectivity and survival, it has the potential to be harnessed into therapeutic tools for gene delivery and antiviral interventions. Furthermore, establishing standards for describing, differentiating, and characterizing viral aggregates is essential to assist studies in this rapidly evolving and expanding scientific field. Findings so far suggest that viral aggregation is a dynamic phenomenon with unpredictable outcomes, and as such, several questions remain yet to be answered. Some of them are given below:

- How commonly do aggregates of pandemic/epidemic/endemic strains of viruses occur in different environments, such as inside a host cell versus a wastewater treatment plant?
- Are there any genetic determinants of viral aggregation? What factors, genetic and otherwise, influence and distinguish the formation of different kinds of viral aggregates, for instance, vesicle-enclosed viral aggregates versus virus–virus binding aggregates versus aggregates formed by virus binding to other surfaces/molecules?
- Does the nature of viral aggregates determine their fate regarding immune evasion and clearance? For instance, vesicle-enclosed viral aggregates show enhanced immune evasion. In contrast, aggregates formed by antibodies are more potent immune stimuli triggering enhanced opsonization and immune clearance.
- How does viral aggregation influence different events of an infectious viral life cycle, including viral adhesion, entry, replication, assembly, and release? What molecular and cellular factors/mechanisms drive those outcomes? Is aggregation conditional on any stage of the viral life cycle?
- How does viral aggregation influence the infectivity and virulence of different viral species or even different strains of the same viral species? Are there aggregation patterns exhibited by viral strains/species that can be traced back to the similarities and differences in their structural/genetic makeup?
- How does aggregation contribute to the viral fitness, diversity, and evolution landscape?
- Can we develop model systems to study viral aggregation? Can we induce viral aggregation in vitro, in vivo, and ex vivo to modulate infectivity, virulence and neutralization?
- How does viral aggregation influence the kinetics and efficiency of viral vectors in gene therapy?

## Figures and Tables

**Table 1:** Microenvironments of different animal-infecting viruses either collected from biological sources or propagated in laboratories and the corresponding EM image of viral aggregation. Scale bars are indicated wherever possible. Panels *a*, *b* and *c* republished with permission from [28]. Copyright 1965 [29]. Copyright 2016 [6]. Copyright 1981, respectively. Panels *d*, *e*, and *f* republished with permission from [7]. Copyright 1985. Panels *g*, *h*, and *i* republished with permission from [30]. Copyright 1993 [5]. Copyright 1977 [31]. Copyright 1985, respectively. All permissions are conveyed through Copyright Clearance Center, Inc.

Virus	Family	Source	Microenvironment of Virus and Aggregating Condition	Ref.	Image
Vaccinia	Poxiviridae	Virus propagated in Earle’s L cells in vitro	Purified virus particles were resuspended in PBS.	[28]	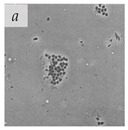
Human adenovirus 2	Adenoviridae	Virus propagated in A549 cells in vitro	Cell associated virus (CAV) particles were resuspended in chlorine demand-free (CDF) grade water.	[29]	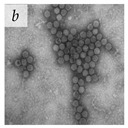
Adenovirus	Adenoviridae	Virus present in fecal specimens of patients with gastroenteritis.	Fecal samples with virus particles were diluted in PBS.	[6]	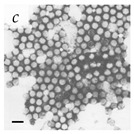 100 nm
Rotavirus	Reoviridae	Virus present in fecal specimens of patients with gastroenteritis.	Fecal samples with virus diluted in water. Image shows aggregates of Rotavirus inside membranes.	[7]	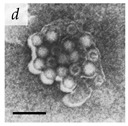 200 nm
Parvovirus	Parvoviridae	Virus present in fecal specimens of patients with gastroenteritis.	Fecal samples with virus diluted in water. Image shows aggregates of Parvovirus inside membranes.	[7]	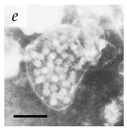 100 nm
Norwalk virus	Caliciviridae	Virus present in fecal specimens of patients with gastroenteritis.	Fecal samples with virus diluted in water. Image shows three Norwalk virus particles associated with a fuzzy membranous element.	[7]	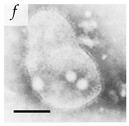 100 nm
Poliovirus	Picornaviridae	Virus-infected Caco-2 cells (MOI = 1)	Arrowhead shows aggregate of poliovirus within an intracellular vesicle of infected Caco-2 cells observed at 16 hpi.	[30]	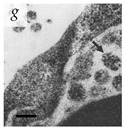 200 nm
Reovirus	Reoviridae	Virus propagated in L cells in vitro	Purified virus particles were diluted in buffers of different pH. Aggregation was observed in buffer with low pH which was reversible when returned to neutral pH.	[5]	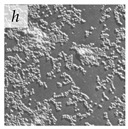
West Nile Virus	Picornaviridae	Virus propagated in Vero cells in vitro.	Aggregate of WNV observed after binding with P388D1 cells for 2 h at 0 °C.	[31]	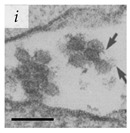 100 nm

**Table 2:** Effects of viral aggregation on the life cycle and pathogenesis of different animal-infecting viruses.

Virus	Genetic Material	Envelope	Family	Size (nm)	Effect of Aggregation on Infection Cycle	Reference
**Baculovirus**	DNA	Enveloped	*Baculoviridae*	200–450	Co-transmission of multiple viral genomes leading to maintenance of genetic diversity [78], enhanced viral protection [79]	[78,79]
**Coronavirus**	RNA	Enveloped	*Coronaviridae*	80–120	Correlated with loss of viral infectivity although not determined as the only cause	[77]
**Echovirus type 4**	RNA	Non-enveloped	*Picornaviridae*	30	Enhanced protection against neutralizing antibodies	[26]
**Enterovirus**	RNA	Non-enveloped	*Picornaviridae*	30	Enhanced protection against neutralizing antibodies [8,26], enhanced infectivity [8]	[8,26]
**Hepatitis A Virus**	RNA	Non-enveloped	*Picornaviridae*	27	Viral aggregates inside host-derived membranes showed enhanced infectivity and resistance against antibodies	[74]
**Human Immunodeficiency Virus**	RNA	Enveloped	*Retroviridae*	120	Tetherin-induced viral aggregates showed reduced infectivity due to impairment of their fusion capabilities [75], enhanced cell-to-cell transfer either by mediating the accumulation of virions on the cell surface or by regulating the integrity of the virological synapse [80]	[75,80]
**Human T-lymphotropic Virus**	RNA	Enveloped	*Retroviridae*	120	Facilitated attachment of virus to target cell surface	[81]
**Influenza A Virus**	RNA	Enveloped	*Orthomyxoviridae*	80–120	Enhanced infective capacity when aggregated by nucleohistones [3], enhanced opsonization and uptake by neutrophils when aggregated by collectins, defensins, or antiviral peptides [76,82,83], decrease in viral uptake and replication by host cells [84]	[3,76,82,83,84]
**Poliovirus**	RNA	Non-enveloped	*Picornaviridae*	30	Aggregates formed in low pH showed decrease in infectious viral titer [32,85] and promoted coinfection that correlated with the mutation frequency and rescue of heavily mutagenized viruses [85]. Vesicle-enclosed viral aggregates showed non-lytic release, enhanced viral spread in vitro and pathogenicity in vivo [86]	[32,85,86]
**Vaccinia virus**	DNA	Enveloped	*Poxvirus*	250–360	Enhanced viral survival via increase in cellular MOI	[28,57]
**Rotavirus**	RNA	Non-enveloped	*Reoviridae*	55–70	Vesicle-enclosed aggregates showed enhanced infectivity in vitro and in vivo by overcoming replication barriers associated with low MOI	[9]
**Vesicular Somatitis Virus**	RNA	Enveloped	*Rhabdoviridae*	70	Co-transmission of multiple viral genomes to same cells [43], saliva-induced viral aggregates showed enhanced viral fitness via increase in per capita progeny production [73]	[43,73]
**West Nile Virus**	RNA	Enveloped	*Flaviviridae*	40–65	Slower uptake and phagocytosis by macrophage-like cells	[31]

**Table 3:** Aggregation of Influenza A virus (IAV) by different biomolecules. Panels *a*, *c*, *d* and *e* republished with permission from [84]. Copyright 2015 [116]. Copyright 2018 [110]. Copyright 2007 [117]. Copyright 2011, respectively; permissions conveyed through Copyright Clearance Center, Inc. Panel *b* republished with permission from [118]. Copyright 2018; permission conveyed through Creative Commons Attribution License.

IAV Strain	Aggregating Factor and Conditions	Ref.	Image
H3N2 A/Philippines/2/82	Arginine-rich histone protein (H4)	[84]	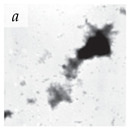
H3N2 A/Philippines/2/82	β-amyloid peptide (βA22-42), which is a 19-amino acid long fragment of the Alzheimer-associated β-amyloid transmembrane precursor protein	[118]	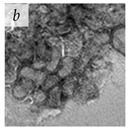
H3N2 A/X-31	IgG antibodies	[116]	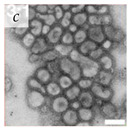
H1N1 A/PR/8/34	Mouse serum with complement proteins and virus-specific antibodies	[110]	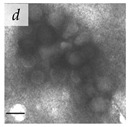
H1N1 A/PR/8/34	EB (Entry Blocker) antiviral peptide derived from fibroblast growth factor 4	[117]	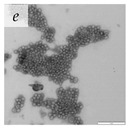

## Abbreviations

The following abbreviations are used in this manuscript:

AAV	Adeno-associated virus
AWOL	Autophagosome-mediated exit without lysis
EB	Entry blocker
EM	Electron microscopy
EMV	Extracellular microvesicles
EV	Extracellular vesicle
FGF-4	Fibroblast growth factor-4
GCXV	Guaico CuleX virus
HA	Hemagglutinin
HAV	Hepatitis A virus
HIV	Human immunodeficiency virus
IAV	Influenza A virus
MOI	Multiplicity of infection
MSD	Mean-squared displacement
MVB	Multivesicular body
NA	Neuraminidase
NET	Neutrophil extracellular trap
OBs	Occlusion bodies
PS	Phosphatidylserine
RSV	Respiratory syncytial virus
TMV	Tobacco mosaic virus
VSV	Vesicular stomatitis virus
